# Associations between weekly maternal exposure to ambient particulate matter and congenital heart disease

**DOI:** 10.3389/fpubh.2025.1627125

**Published:** 2025-12-09

**Authors:** Sun Li, Shao Yan, Gong Tian, Peng Xiaoju, Zhou Fanjie, Wu Qianlan

**Affiliations:** Suzhou Municipal Hospital, Suzhou Women and Childrens Health Hospital, The Affiliated Suzhou Hospital of Nanjing Medical University, Suzhou, China

**Keywords:** ambient particulate matter, gestational exposure, congenital heart defects, susceptible exposure windows, Suzhou

## Abstract

**Background:**

The association between maternal exposure to air pollution and congenital heart diseases (CHDs) has garnered increasing attention. However, investigations into critical weekly specific exposure windows for CHDs remain limited. This study evaluated the relationship between maternal ambient particulate matter exposure throughout the entire pregnancy period and the risk of CHDs, as well as explored critical weekly specific exposure windows.

**Method:**

Based on the Jiangsu Maternal and Child Health Monitoring Manual and the Environmental Health Department of Suzhou CDC, 19,914 pregnant women with complete hospital delivery records between 2021 and 2023 were included, and the concentration data for five air pollutants (PM_10_, PM_2.5_, NO_2_, CO, and SO_2_) in Suzhou City from 2020 to 2023 were obtained in this study. Distributed lag models (DLMs) incorporated in Cox proportional-hazards models were applied to assess the exposure-lag-response relationship between the weekly average exposure levels during pregnancy and the risk of CHDs, identifying the refined exposure windows.

**Results:**

A 10 μg/m^3^ increase in maternal exposure to PM_10_ was positively associated with CHDs during gestational weeks 5–12 and weeks 18–22 (*p* < 0.05). The strongest association was observed in gestational weeks 5 and 6 (HR, 1.010; 95% CI, 1.000–1.020; HR, 1.010; 95% CI, 1.002–1.017). The association between maternal exposure to PM_2.5_ and the risk of CHDs was not observed during all gestational weeks.

**Conclusion:**

Our study results demonstrate that exposure to high concentrations of PM_10_ increases the risk of CHDs, with susceptible exposure windows identified as gestational weeks 5–12 and weeks 18–22. Our study provides further evidence supporting the association between maternal exposure to ambient particulate matter and CHDs while also identifying critical windows. However, further studies are required to confirm these findings.

## 1 Introduction

Congenital heart diseases (CHDs), commonly defined as a structural abnormality of the heart or great vessels present at birth, are the most common congenital disease among newborns. The average prevalence rate of CHD worldwide is approximately 9.4‰, with the highest prevalence rate in Asia (1). In China, there were 12.7 cases of CHD per 1,000 live births in 2019, with a trend of increasing prevalence year by year (2). The incidence in Suzhou was consistent with the national trend in recent years. In 2022, the incidence of CHDs in Suzhou was 70/10,000, accounting for 45.14% of total perinatal birth defects and recognized as a major cause of neonatal death due to birth defects.

Embryonic development is one of the critical windows in the growth and development process, and maternal exposure to air pollutants during pregnancy cannot be overlooked in the development of CHDs. Various health hazards associated with air pollution have become increasingly apparent. Since the late 1990s, the impact of air pollution on adverse pregnancy outcomes has garnered increasing attention (3, 4).

Ambient particulate matter, especially PM with an aerodynamic diameter of 10 μm (PM_10_) and PM with an aerodynamic diameter of 2.5 μm (PM_2.5_), is characterized by its small size, large specific surface area, and long residence time in the air. These particles tend to accumulate various toxic and harmful substances, making them a primary threat to human health (5). Animal experiments have indicated that particulate matter may induce heart defects through oxidative stress, DNA damage, and changes in molecular signals or epigenetic events (6, 7). However, the conclusions from existing population studies were not consistent, potentially due to the complex composition of air pollutants, such as different particle sizes, differences in demographic characteristics among study populations, age distribution, and varying levels of population susceptibility. Although the existing research findings were not entirely consistent, a growing body of epidemiological literature has suggested that PM_10_ and PM_2.5_ exposure may result in adverse birth outcomes, including CHD (8, 9). An increasing number of scholars have focused their attention on critical windows for maternal air pollutant exposure and adverse birth outcomes (10). Over the last decade, numerous domestic and international studies on susceptibility windows have divided pregnancy into several relatively long exposure periods, such as the first, second, and third trimester (11, 12). Other epidemiological and clinical evidence suggested that weeks 3–8 of pregnancy were the developmental period for most human organs and were susceptible to environmental exposure or teratogens; consequently, many studies have selected weeks 3–8 of pregnancy as the exposure window (13–15). Subsequently, an increasing number of researchers considered that the division of longer exposure periods may overlook potential critical windows, and they are beginning to attempt to further refine the temporal scale of pollutant exposure to more accurately determine the critical exposure window period (16, 17). However, few studies in China have effectively evaluated the correlation between weekly average ambient particulate matter exposure throughout pregnancy and CHDs. Therefore, it is urgent to clarify the effects of maternal ambient particulate matter exposure on CHDs.

With the accelerating urbanization process in Suzhou Industrial Park, air pollution issues are expected to persist for a considerable period. Currently, eugenics and quality-oriented childbearing are particularly important in this new fertility situation. The main objective of this study is to estimate the association between maternal exposure to ambient particulate matter and CHDs, as well as to identify specific windows using distributed lag models (DLMs) incorporated in Cox proportional-hazards models. The findings of this study could provide scientific evidence for air pollution prevention and control in Suzhou City and simultaneously offer higher-quality perinatal care for pregnant women at the same time.

## 2 Methods

### 2.1 Study population

**Figure 1:**
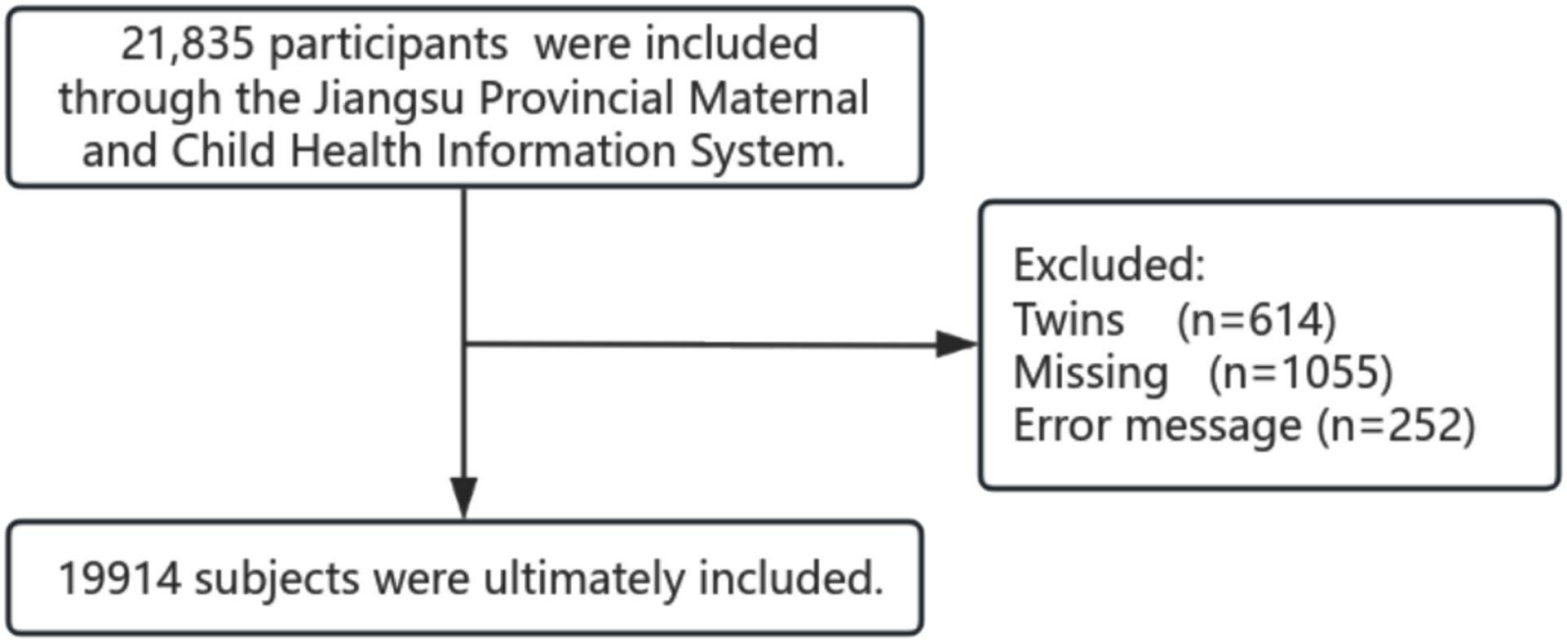
Participant selection flowchart.

Our data on pregnancy and childbirth were extracted from the birth defect monitoring system in the Jiangsu Maternal and Child Health Monitoring Manual and included birth date, gestational age at birth, infant sex, maternal age, gravidity, and parity. Birth information, such as infant sex and birth weight, was recorded by medical personnel at midwifery agencies. We included mothers living in Suzhou Industrial Park during pregnancy according to their maternal residential address and excluded those with multiple pregnancies. The inclusion criteria were as follows: Participants were required to be permanent residents of Suzhou Industrial Park who had completed their first-trimester (1–13^+6^weeks) registration there. Additionally, they must have received at least one first-trimester (1–13^+6^weeks), 2 s-trimester (14–27^+6^weeks), and three third-trimester (≥28 weeks) prenatal check-ups in Suzhou and have delivered at a maternity hospital within the city. A total of 19,914 pregnant women were recruited between 2021 and 2023. The date of conception was defined as the 14th day after the date of the last menstruation, and the first trimester (1–13^+6^weeks), second trimester (14–27^+6^ weeks), and third trimester (≥28 weeks) were categorized accordingly. This study was approved by the Ethics Committee of Suzhou Municipal Hospital (approval No. IEC-C-008-a07-V1.0). In the final database, only the numbers of mothers and newborns were listed. All personal information was kept confidential (Figure 1).

### 2.2 Congenital heart disease data

In 2019, Suzhou City launched a CHD screening project in all medical institutions with obstetric service qualifications. Newborns were screened by neonatologists using the dual-indicator method (cardiac auscultation and percutaneous pulse oximetry screening) within 6–72 h after birth. Any positive screening for either indicator resulted in a positive screening outcome, and echocardiography was performed for further diagnosis. Subjects with abnormalities confirmed by echocardiography were enrolled as CHD cases in this study. All information on CHD screening and diagnosis for children in Suzhou was entered into the Jiangsu Maternal and Child Health Information System.

### 2.3 Environmental exposure data

The daily concentrations of ambient air pollution data collected in this study were provided by the Environmental Health Department of the Suzhou CDC, based on the average of two air monitoring stations in the Suzhou Industrial Park. Monitored pollutants included nitrogen dioxide (NO_2_), sulfur dioxide (SO_2_), carbon monoxide (CO), particulate matter with aerodynamic diameters smaller than 10 μm (PM_10_), and 2.5 μm (PM_2.5_). In this study, weekly air pollution levels during pregnancy were estimated for each pregnant woman based on daily exposure. The concentrations of the pollutants were expressed as μg/m^3^.

### 2.4 Statistical methods

The database was analyzed using the Statistical Package for the Social Sciences (SPSS, Chicago, Illinois, USA), version 17.0, and R 4.3.1. SPSS was used for general description and the chi-square test. Continuous variables are represented as mean ± standard deviation, and categorical variables are presented as numbers and frequencies. Distributions of absorbable particulate matter exposure during different gestations are presented as quartiles and interquartile ranges (IQR). Pearson’s correlations between different air pollutants during the three trimesters were calculated. The chi-square test was used to examine statistically significant differences in sociodemographic characteristics between infants with CHDs and other infants. A *p* value of < 0.05 was considered statistically significant. We employed Cox proportional hazard models to analyze the association between exposure during different trimesters and the risk of congenital heart defects for each 10 mg/m^3^ increment in PM_2.5_ and PM_10_. Subsequently, we applied distributed lag models (DLMs) incorporated into Cox proportional-hazards models to assess the exposure-lag-response relationship between the weekly average exposure levels of pollutants during pregnancy and the risk of CHDs, identifying the refined exposure windows. The results are presented as the change in outcome for every 10 mg/m^3^ increase in the concentration. The framework of DLMs was based on a “coss basis” function. The lag distribution for PM was modeled as natural cubic splines with optimal degrees of freedom (df) of 4 based on the minimum Akaike Information Criterion (AIC), assuming that the exposure-response association varies smoothly across weeks. All covariates with statistical significance in the chi-square test were sequentially included.

## 3 Results

### 3.1 Descriptive statistics

A total of 19,914 pregnant women were recruited, and 503 newborns tested positive for CHD screening. Statistically significant differences were observed in the distribution of delivery modes and birth weights between children with congenital heart defects and those without (*p* < 0.05). There were no statistically significant differences in maternal age, number of pregnancies, total previous live births, season of conception, or infant gender, which were no longer included in the Cox proportional hazard models and distributed lag linear models (Table 1).

**Table 1:** Demographic characteristics of the subjects from the present study.

Characteristics		Congenital heart defects		*χ* ^2^	*P*-value
		Yes *n* (%)	No *n* (%)		
Neonatal gender	Male	273 (54.3)	10,035 (51.7)	1.304	0.254
	Female	230 (45.7)	9,376 (48.3)		
Birth weight (g)	<2,500 g	33 (6.6)	610 (3.1)	18.621	<0.001^*^
	2,500–4000 g	440 (87.5)	17,713 (91.3)		
	≥4,000 g	30 (5.9)	1,088 (5.6)		
Number of pregnancies	1	195 (38.8)	7,663 (39.5)	1.561	0.458
	2	140 (27.8)	5,747 (29.6)		
	≥3	168 (33.4)	6,001 (30.9)		
Mode of delivery	Cesarean section	249 (49.5)	7,295 (37.6)	29.610	<0.001^*^
	Vaginal delivery	254 (50.5)	12,116 (62.4)		
Total previous live births	0	261 (51.9)	10,566 (54.4)	1.374	0.503
	1	213 (42.3)	7,840 (40.4)		
	≥2	29 (5.8)	1,005 (5.2)		
Season of conception	Spring (March–May)	114 (22.7)	5,060 (26.1)	5.432	0.143
	Summer (June–August)	117 (23.3)	4,770 (24.5)		
	Autumn (September–November)	132 (26.2)	4,885 (25.2)		
	Winter (December–February)	140 (27.8)	4,696 (24.2)		
Maternal age	<20	2 (0.4)	79 (0.4)	0.109	0.947
	20–35	419 (83.3)	16,273 (83.8)		
	≥35	82 (16.3)	3,059 (15.8)		

^*^p < 0.05.

### 3.2 Maternal exposure to air pollutants

The average exposure levels of PM_10_ during the first trimester, second trimester, and third trimester for the pregnant women in this study were 47.1 μg/m^3^, 46.7 μg/m^3^, and 47.2 μg/m^3^, respectively, and the average maternal PM_2.5_ exposure during the entire pregnancy period was 27.8 μg/m^3^, 27.4 μg/m^3^, and 27.5 μg/m^3^, respectively. During our research, the minimum daily PM_10_ and PM_2.5_ concentrations were 22.2 μg/m^3^ and 12.0 μg/m^3^, and the maximum daily PM_10_ and PM_2.5_ concentrations were 94.2 μg/m^3^ and 57.3 μg/m^3^_._ Distributions of PM_10_ and PM_2.5_ were presented by quartile and interquartile range (IQR) averaged during different trimesters of pregnancy (Supplementary Table 1). Air pollutants are significantly associated with each other during pregnancy. Other pollutants were controlled as confounding factors when analyzing pollutants in subsequent models (Supplementary Table 2).

### 3.3 Association between maternal absorbable particulate matter exposure and CHDs

Table 2 presents the HRs of CHDs associated with every 10 μg/m^3^ increase in PM_2.5_ and PM_10_ over different trimesters and the entire pregnancy period. In the crude model, every 10 μg/m^3^ increase in PM_10_ was positively associated with the risk of CHDs during the first trimester (HR, 1.009; 95% CI, 1.002–1.016). In contrast, PM_10_ was significantly negatively correlated to CHDs in the third trimester (HR, 0.992; 95% CI, 0.986–0.999). However, these significant correlations were not observed in the models adjusted for confounding factors. No significant effect of PM_2.5_ on CHDs was observed in either the crude model or the adjusted model during each pregnancy period.

**Table 2:** Crude and adjusted hazard ratios of maternal exposure to PM_10_, PM_2.5_, and congenital heart disease in different gestation.

Exposure windows	PM_10_		PM_2.5_	
	Crude model (95%CI)	Adjusted model (95%CI)	Crude model (95%CI)	Adjusted model (95%CI)
First trimester	1.009(1.002–1.016)^*^	0.997(0.989–1.011)	1.01(1.000–1.020)	0.997(0.981–1.013)
Second trimester	1.004(0.997–1.011)	1.01(0.999–1.021)	1.004(0.994–1.014)	1.01(0.992–1.028)
Third trimester	0.992(0.986–0.999)^*^	0.997(0.986–1.008)	0.987(0.977–0.997)	0.989(0.972–1.007)
Entire trimester	1.009(0.995–1.024)	1.011(0.987–1.037)	1.009(0.980–1.039)	0.996(0.945–1.050)

^*^HRs and 95% CIs were calculated per 10 μg/m^3^ increase in PM_10_ and PM_2.5_; the crude model was adjusted for no covariates; and the adjusted model was adjusted for birth weight, mode of delivery, and for the effects of other air pollutants in the same time window.

**Figure 2:**
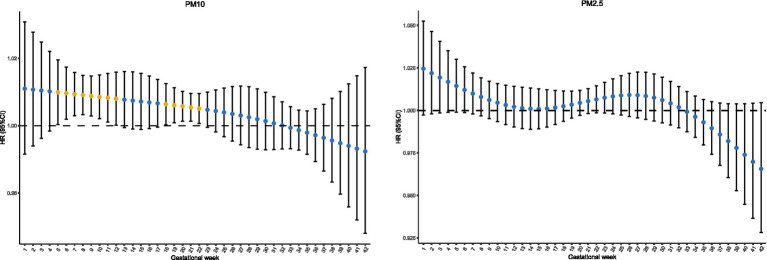
Associations of weekly PM10 and PM2.5 exposure during gestational weeks with risks of CHDs.

The results of distributed lag models (DLMs) incorporated in Cox proportional-hazards models indicated that the weekly average exposure levels of PM_10_ during the first and second pregnancy trimesters were associated with the risk of CHDs after adjusting for confounding factors. The susceptible window for PM_10_ exposure in relation to CHDs was identified as weeks 5–12 and weeks 18–22 of pregnancy (*p* < 0.05), with the strongest association observed in the 5th and 6th gestational weeks (HR, 1.010; 95% CI, 1.000–1.020; HR, 1.010; 95% CI, 1.002–1.017) (Figure 2). However, no association was found between maternal exposure to PM_2.5_ and the risk of CHDs across all gestational weeks.

### 3.4 Diagnostic testing of models

We conducted comprehensive diagnostic tests to evaluate the statistical properties and adherence to model assumptions. Autocorrelation analysis indicated no significant temporal dependence in the residuals of either model. The Ljung–Box test *p*-values for PM_2.5_ and PM_10_ were 0.311 and 0.339, respectively—both well above the 0.05 significance level—and autocorrelation coefficients at all lags were close to zero, supporting the assumption of residual independence. In the proportional hazards assumption test, the PM2.5, PM10, and delivery mode variables all satisfied this assumption (*p*-values: 0.374, 0.418, and 0.415), whereas the weight category variable did not (*p*-values: 0.00014 and 0.00018), suggesting a time-varying effect of weight on health outcomes. Residual analysis showed that Martingale residuals for both models were randomly distributed around zero, deviance residuals exhibited no abnormal patterns, and smoothed survival curves indicated a good overall model fit. For multicollinearity, variance inflation factor (VIF) values ranged from 1.445 to 5.405 in the PM_2.5_ model and from 1.588 to 5.146 in the PM_10_ model—all well below the threshold of 10—confirming no severe multicollinearity and supporting the stability and reliability of the parameter estimates. In summary, both PM_2.5_ and PM_10_ distributed lag non-linear models demonstrated sound statistical performance, providing accurate and reliable assessments of the associations between air pollutant exposure and health outcomes.

## 4 Discussion

Although genetic factors play an important role in the development of CHD, studies have shown that only approximately 15% of CHD cases can be attributed to genetic factors, while the remaining 85% are influenced by the interaction of multiple factors, including genetics and the environment (18). Our study demonstrated that maternal exposure to low-level ambient air pollution (46.5 μg/m^3^ for PM_10_ and 27.3 μg/m^3^ for PM_2.5_) during the entire pregnancy in Suzhou Industrial Park was lower than annual average maternal exposure concentrations in other cities like Tianjin, Shanghai, Hefei, and Guangzhou (16, 17, 19, 20). However, this exposure concentration was significantly higher than that in some cities abroad, such as Brisbane and Victoria (21, 22). Individual exposure to PM_10_ (mean ± SD) was highest in the third trimester, while individual exposure to PM_2.5_ (mean ± SD) was highest in the first trimester. Compared to our previous study on the analysis of maternal exposure to air pollutants during pregnancy from 2015 to 2019 (23), the overall exposure concentration of particulate matter during pregnancy in this study has decreased, indicating that with the implementation of various measures to combat haze, the air pollution situation in Suzhou City has improved to some extent, and there has been a general trend of reduction in the concentrations of PM_10_ and PM_2.5_.

In the present study, we observed that weekly exposure to PM_10_ increased the risk of CHDs. The susceptible windows were identified as the 5th–12th weeks and 18th–22nd weeks of pregnancy, with the strongest association observed during the 5th and 6th gestational weeks. We also evaluated the association by trimester, but no significant association between either PM_10_ or PM_2.5_ and the risk of CHDs was observed across all the aforementioned exposure windows. Our study results further revealed that the sensitivity calculated based on exposure levels during different pregnancy trimesters was lower than that calculated on a weekly specific scale. The first susceptibility window was identified during early pregnancy (weeks 5–12). This finding strongly suggests that exposure to ambient PM10 during the initial and active phases of morphological heart development may be a significant risk factor. The second susceptibility window emerged during mid-pregnancy (weeks 18–22), indicating that pollutant exposure might still exert disruptive effects during the late-shaping processes of the heart. Previous studies on the association between environmental pollutants and adverse birth outcomes have reached such conclusions (17, 19). At present, a significant amount of studies have focused on exposure windows measured on a monthly or trimester scale, and some results are similar to ours. For example, birth cohort studies in Foshan and Wuhan found no association between PM_2.5_ and PM_10_ exposure, and the risk of CHD (15, 24). To the best of our knowledge, only a few studies have evaluated weekly specific associations between maternal ambient particulate matter exposure and CHDs. Moreover, the results of the sensitive exposure gestational weeks obtained from these studies were not consistent. A study in Hefei evaluated the associations of weekly air pollution exposure and CHDs, results showed that the susceptibility windows of PM_2.5_ (weeks 20–26), PM_10_ (weeks 0–2 and weeks 25–29 of pregnancy), while the strongest effects on CHDs were observed in week 22 (RR = 1.034, 95% CI: 1.007–1.062) and week 0 (RR = 1.081, 95% CI: 1.02–1.146), respectively (16). Zhang et al. in Wuhan reported a significant association between PM_2.5_ exposure during weeks 7–10 of pregnancy and the risk of VSD^14^. Many early studies suggested that weeks 0–8 were a sensitive window period for the risk of CHDs associated with PM_10_, which partially aligned with the sensitive window period we derived from our study (25–28). Regrettably, our study found no significant correlation between PM_2.5_ and the risk of CHDs; one possible reason was that we did not classify CHDs into specific subtypes. A considerable number of studies have suggested that the association between particulate matter and specific congenital heart subtypes varies significantly. Padula et al. clarified that PM_10_ was associated with pulmonary stenosis (PS) and ventricular septal defect (VSD), while PM_2.5_ was associated with transposition of the great arteries (TGA) and inversely associated with VSD and atrial septal defect (ASD) (28). The inconsistency in associations and sensitive windows across different study results may be due to the following reasons: First, the exposure concentrations of pollutants varied from place to place, and evidence suggests that lower exposure levels may limit the strength of the corresponding associations (29). Second, demographic characteristics, immunity factors, and other variables may differ among the study population groups. Finally, there was heterogeneity in the assessment methods for environmental pollutant exposure and the diagnosis of CHDs in different studies.

Environmental factors, another crucial risk factor for CHDs, have always been a focus of attention. A study in California highlighted that individuals were susceptible to the interaction effects of genetics and the environment (30). However, the specific mechanisms of ambient particulate matter impacts on CHDs remain to be fully elucidated. In recent years, advancements in genomic detection technologies and model animal experimentation have propelled studies on gene–environment interactions, providing valuable insights into the mechanisms underlying CHD development. Many studies have demonstrated that placental DNA methylation and RNA expression are closely linked to numerous prenatal exposures, such as air pollution, metals, and maternal smoking, as well as infant and childhood health outcomes (31). This highlights the potential role of epigenetic modifications in mediating the effects of environmental factors on fetal development. Animal experiments showed that particulate matter induced cardiac defects through pathways involving oxidative stress, DNA damage, and changes in molecular signaling or epigenetic events (6). Zebrafish embryo experiments have shown that PM_2.5_ can cause cardiac developmental malformations by activating the aromatic hydrocarbon receptor (AHR), mediating EOM-induced oxidative stress, and resulting in DNA damage and apoptosis (32). These findings underscore the potential mechanisms by which particulate matter exposure can disrupt normal cardiac development. Embryology studies suggest that 3–8 weeks during pregnancy are critical for organ development and are susceptible to environmental exposure or teratogens, indicating that early pregnancy may represent a critical window for exposure to environmental pollutants. Further experimental studies are needed to provide mechanistic support for the epidemiological study results.

Our study has several strengths. To the best of our knowledge, this study is one of the few to evaluate weekly specific associations between PM_2.5_ and PM_10_ exposure, and the risk of congenital heart defects. Therefore, our findings provide new evidence to identify more refined susceptible exposure windows during pregnancy. Despite these strengths, this study has some limitations. Because of the inability to obtain information such as the distance between study subjects and air monitoring stations, exposure levels were reliant on fixed-site monitoring data, which may not capture true individual-level exposure variation. We estimate that this likely results in exposure misclassification, biasing the effect estimates (e.g., hazard ratios) for the associations between PM2.5 and health outcomes toward the null hypothesis and potentially leading to an underestimation of the true health effects. Furthermore, existing studies have found that high temperatures and other environmental factors can enhance the adverse effects of air pollutants on heart development; however, environmental confounding factors, such as temperature, humidity, and other types of air pollutants, were not included in this study. At the same time, CHDs encompass a wide variety of subtypes, and there is heterogeneity in the causal relationship between different subtypes and air pollutants. Regrettably, CHDs were not subcategorized into specific subtypes in this study. In future studies, it is necessary to increase the number of experiments and population studies on exposure to air pollutants and specific subtypes of CHDs to gain a deeper understanding of the mechanisms. Because of the limitations in our data collection, we were unable to account for several potential confounding factors, such as genetic issues in parents and the fetus, maternal complications, exposure to secondhand smoke, and alcohol consumption history. Furthermore, collecting individual-level behavioral data (e.g., mobility trajectories and mask-wearing practices) is crucial for enabling a more refined personal exposure assessment in the context of future major public health events.

In summary, distributed lag models (DLMs) incorporated in Cox proportional-hazards models were applied in this study to refine exposure periods and more precisely identify susceptible windows for the effects of PM_2.5_ and PM_10_ exposure on CHDs. The analysis showed that a high level of outdoor PM_10_ was associated with an increased risk of CHDs. Weeks 5–12 and 18–22 during pregnancy were identified as susceptible windows, with the strongest effects of PM_10_ on CHDs observed during weeks 5 and 6 of gestation. This study has potentially important public health implications for formulating intervention measures to reduce CHDs caused by environmental particulate matter pollution.

## Data Availability

The raw data supporting the conclusions of this article will be made available by the authors, without undue reservation.
