# Supplementary material for: Associations between weekly maternal exposure to ambient particulate matter and congenital heart disease
Source: Front Public Health. 2025 Dec 9;13:1627125. doi: 10.3389/fpubh.2025.1627125 (PMC12722514; doi:10.3389/fpubh.2025.1627125)
Supplement: Supplementary file 1 [file Data_Sheet_1.PDF]

Supplementary Material:

Table1 Summary levels of maternal exposure to each pollutant during entire pregnancy periods(μg/m^3)

Exposure windows	Pollutant	Mean ± SD	Minimum	Maximum	P_{25}	P_{50}	P_{75}
first trimester	PM_{10}	47.1±12.6	29.5	74.7	35.3	45.2	57.3
	$PM_{2.5}$	27.8±8.6	15.4	44.2	20.1	27.3	35.2
second trimester	PM_{10}	46.7±13.2	29.5	74.7	34.1	44.4	57.3
	$PM_{2.5}$	27.4±8.6	15.5	43.5	19.6	25.8	35.3
third trimester	PM_{10}	47.2±14.2	22.2	94.2	33.8	44.9	59.8
	$PM_{2.5}$	27.5±9.2	12.0	57.3	18.5	26.0	35.8
entire pregnancy	PM_{10}	46.5±6.3	32.9	64.8	42.4	45.1	51.3
	$PM_{2.5}$	27.3±3.2	18.6	35.2	24.9	28.2	29.7

Table2 The correlation of different air pollutants during diferent gestation

Exposure windows	Air pollutants	PM_{10}	$PM_{2.5}$	NO_2	CO	SO_2
first trimester	PM_{10}	1				
	$PM_{2.5}$	0.873**	1			
	NO_2	0.632**	0.701**	1		
	CO	0.329**	0.445**	0.807**	1	
	SO_2	0.766**	0.707**	0.663**	0.369**	1
second trimester	PM_{10}	1				

	$PM_{2.5}$	0.889**	1			
	NO_2	0.666**	0.754**	1		
	CO	0.359**	0.488**	0.808**	1	
	SO_2	0.632**	0.640**	0.517**	0.359**	1
third trimester	PM_{10}	1				
	$PM_{2.5}$	0.890**	1			
	NO_2	0.690**	0.774**	1		
	CO	0.487**	0.642**	0.859**	1	
	SO_2	0.665**	0.642**	0.532**	0.413**	1
entire trimester	PM_{10}	1				
	$PM_{2.5}$	0.795**	1			
	NO_2	0.365**	0.556**	1		
	CO	0.209**	0.424**	0.936**	1	
	SO_2	0.727**	0.746**	0.837**	0.776**	1

* $p<0.05$ ** $p<0.01$
