# Supplementary material for: Associations between weekly maternal exposure to ambient particulate matter and congenital heart disease
Source: Front Public Health. 2025 Dec 9;13:1627125. doi: 10.3389/fpubh.2025.1627125 (PMC12722514; doi:10.3389/fpubh.2025.1627125)
Supplement: Supplementary file 2 [file Table_1.DOCX]

**Supplementary Material:**

**Table1 Summary levels of maternal exposure to each pollutant during entire pregnancy periods(μg/m^3^)**

| Exposure windows | Pollutant | Mean ± SD | Minimum | Maximum | P_25_ | P_50_ | P_75_ |
| --- | --- | --- | --- | --- | --- | --- | --- |
| first trimester | PM_10_ | 47.1±12.6 | 29.5 | 74.7 | 35.3 | 45.2 | 57.3 |
|  | PM_2.5_ | 27.8±8.6 | 15.4 | 44.2 | 20.1 | 27.3 | 35.2 |
| second trimester | PM_10_ | 46.7±13.2 | 29.5 | 74.7 | 34.1 | 44.4 | 57.3 |
|  | PM_2.5_ | 27.4±8.6 | 15.5 | 43.5 | 19.6 | 25.8 | 35.3 |
| third trimester | PM_10_ | 47.2±14.2 | 22.2 | 94.2 | 33.8 | 44.9 | 59.8 |
|  | PM_2.5_ | 27.5±9.2 | 12.0 | 57.3 | 18.5 | 26.0 | 35.8 |
| entire pregnancy | PM_10_ | 46.5±6.3 | 32.9 | 64.8 | 42.4 | 45.1 | 51.3 |
|  | PM_2.5_ | 27.3±3.2 | 18.6 | 35.2 | 24.9 | 28.2 | 29.7 |

**Table2** The correlation of different air pollutants during diferent gestation

| Exposure windows | Air pollutants | PM_10_ | PM_2.5_ | NO_2_ | CO | SO_2_ |
| --- | --- | --- | --- | --- | --- | --- |
| first trimester | PM_10_ | 1 |  |  |  |  |
|  | PM_2.5_ | 0.873** | 1 |  |  |  |
|  | NO_2_ | 0.632** | 0.701** | 1 |  |  |
|  | CO | 0.329** | 0.445** | 0.807** | 1 |  |
|  | SO_2_ | 0.766** | 0.707** | 0.663** | 0.369** | 1 |
| second trimester | PM_10_ | 1 |  |  |  |  |
|  | PM_2.5_ | 0.889** | 1 |  |  |  |
|  | NO_2_ | 0.666** | 0.754** | 1 |  |  |
|  | CO | 0.359** | 0.488** | 0.808** | 1 |  |
|  | SO_2_ | 0.632** | 0.640** | 0.517** | 0.359** | 1 |
| third trimester | PM_10_ | 1 |  |  |  |  |
|  | PM_2.5_ | 0.890** | 1 |  |  |  |
|  | NO_2_ | 0.690** | 0.774** | 1 |  |  |
|  | CO | 0.487** | 0.642** | 0.859** | 1 |  |
|  | SO_2_ | 0.665** | 0.642** | 0.532** | 0.413** | 1 |
| entire trimester | PM_10_ | 1 |  |  |  |  |
|  | PM_2.5_ | 0.795** | 1 |  |  |  |
|  | NO_2_ | 0.365** | 0.556** | 1 |  |  |
|  | CO | 0.209** | 0.424** | 0.936** | 1 |  |
|  | SO_2_ | 0.727** | 0.746** | 0.837** | 0.776** | 1 |
| * *p*<0.05 ** *p*<0.01 | | | | | | |
